# Supplementary figures and images for: ATAD2 mediates chromatin-bound histone chaperone turnover
Source: eLife. 2026 Jan 20;14:RP107582. doi: 10.7554/eLife.107582 (PMC12818868; doi:10.7554/eLife.107582)

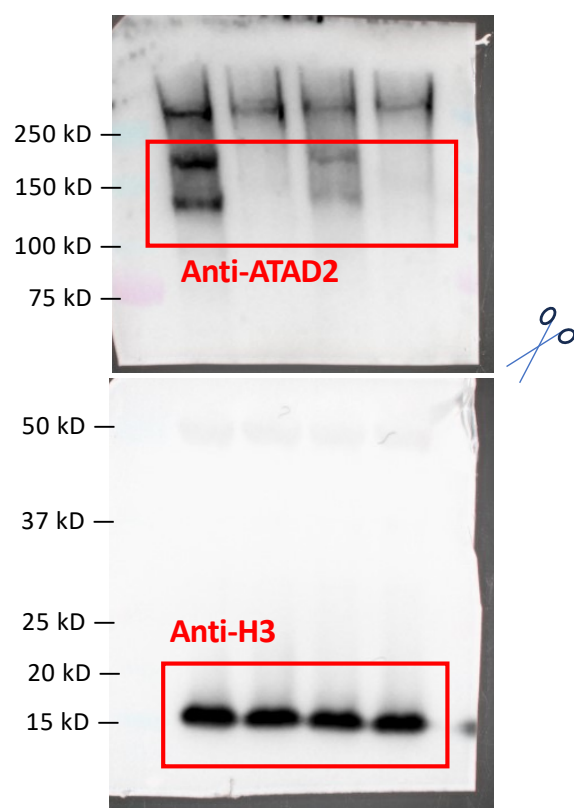

Supplement: Figure 1—source data 1. [file elife-107582-fig1-data1.zip › Figure 1C source data 1/Figure 1C-source data 1.pdf]

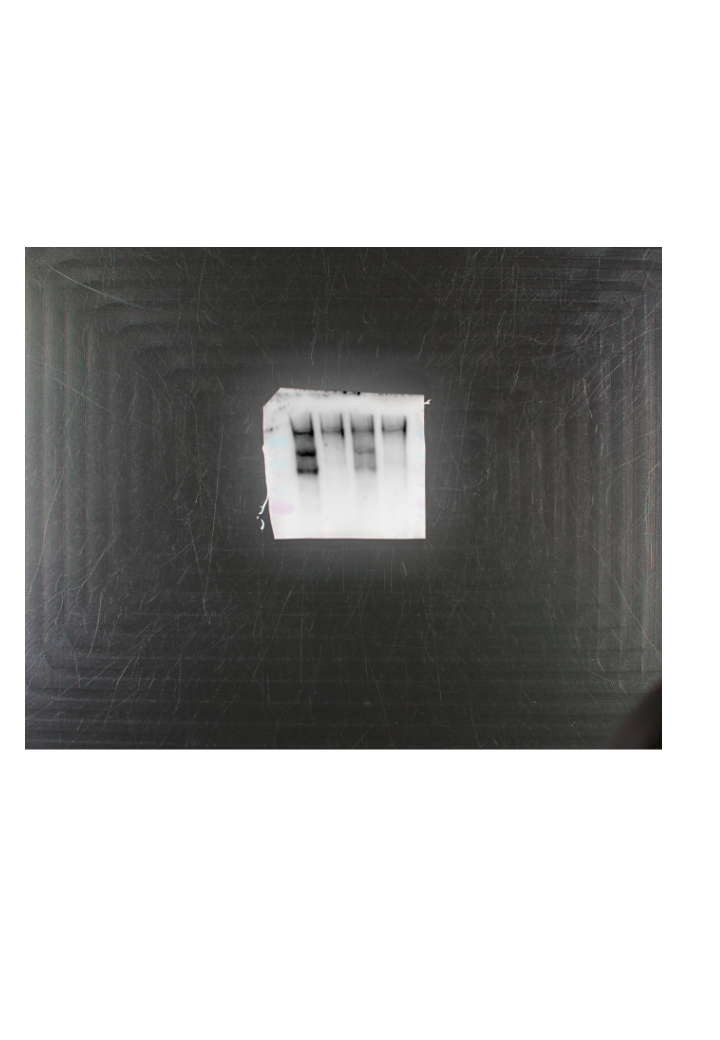

Supplement: Figure 1—source data 2. [file elife-107582-fig1-data2.zip › Figure 1C source data 2/Figure 1C_source data 2_Atad2.tiff]

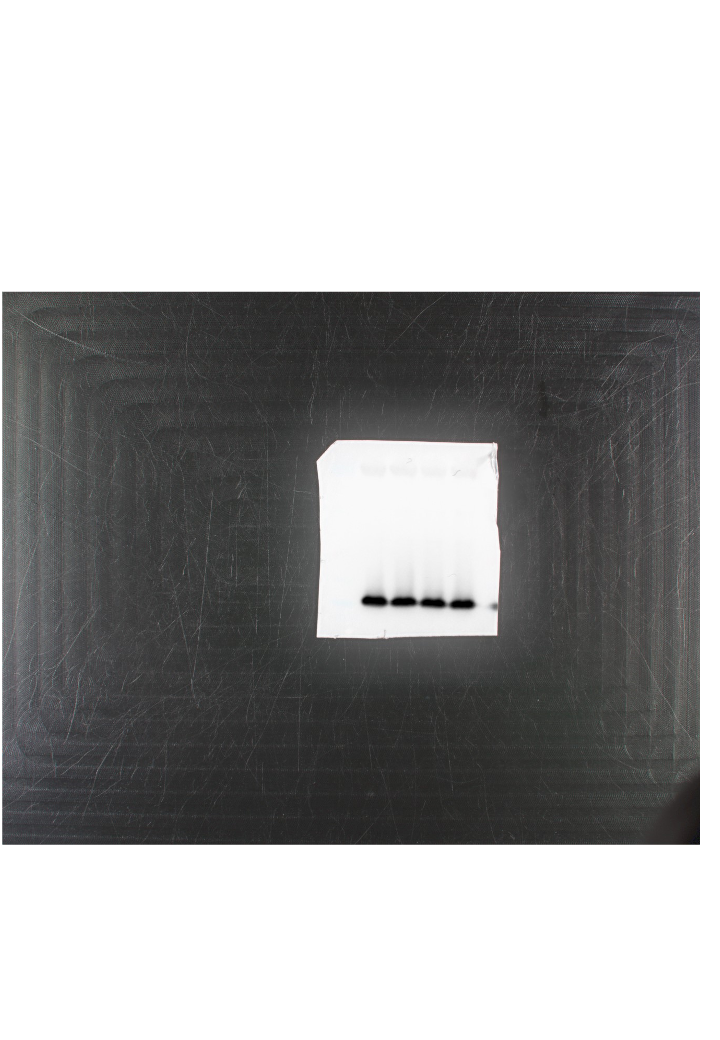

Supplement: Figure 1—source data 2. [file elife-107582-fig1-data2.zip › Figure 1C source data 2/Figure 1C_source data 2_H3.tiff]

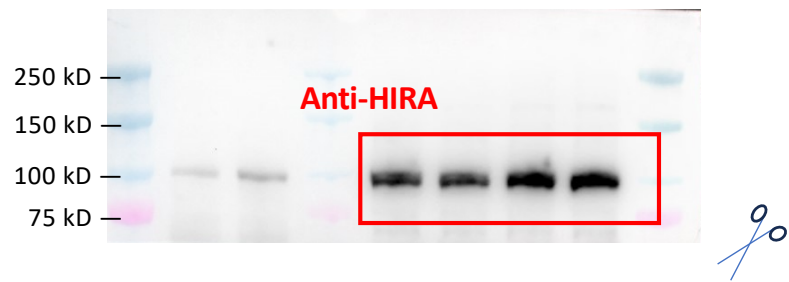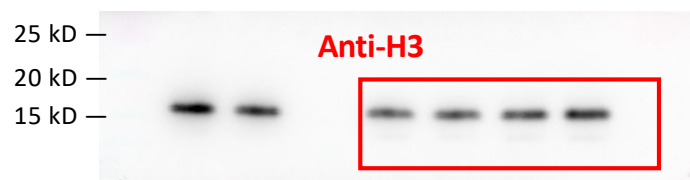

Supplement: Figure 2—source data 1. [file elife-107582-fig2-data1.zip › Figure 2A source data 1/Figure 2A - source data 1.pdf]

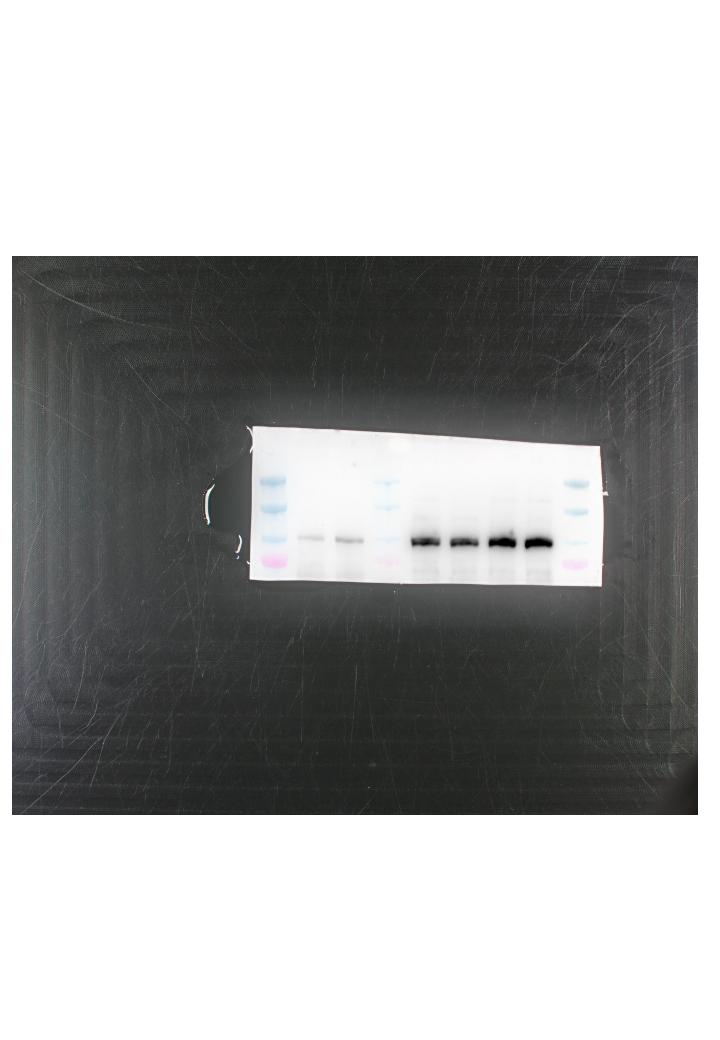

Supplement: Figure 2—source data 2. [file elife-107582-fig2-data2.zip › Figure 2A source data 2/Figure 2A_source data_HIRA.tiff]

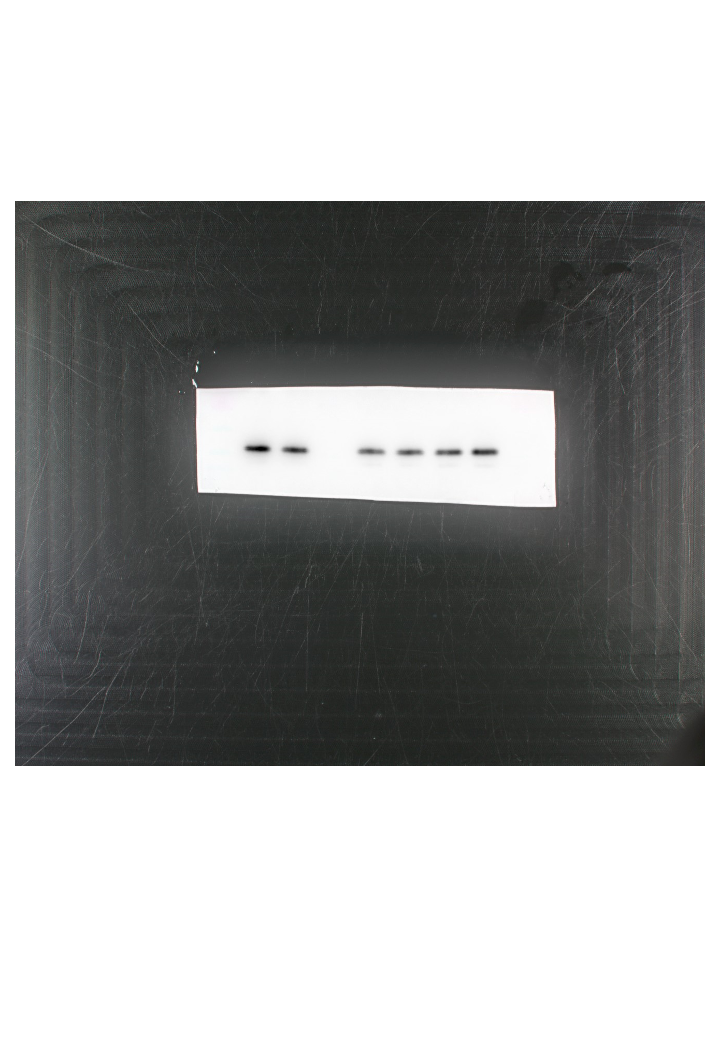

Supplement: Figure 2—source data 2. [file elife-107582-fig2-data2.zip › Figure 2A source data 2/Figure 2A_source data_H3.tiff]

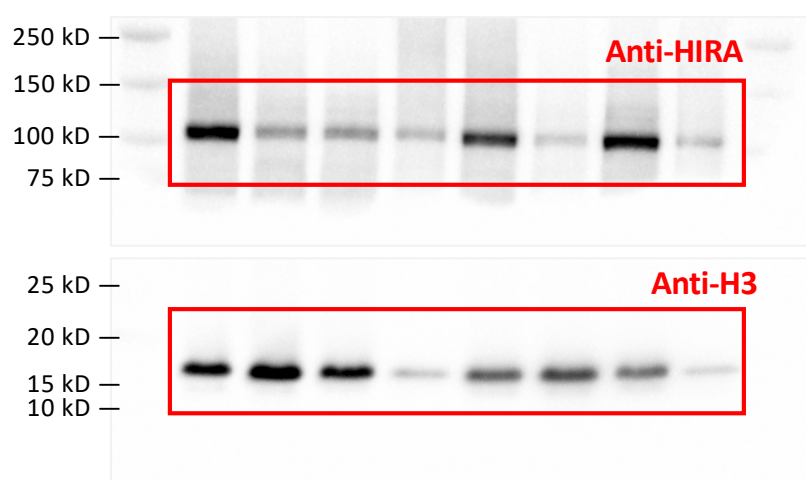

Supplement: Figure 2—source data 3. [file elife-107582-fig2-data3.zip › Figure 2B source data 1/Figure 2B - source data 1.pdf]

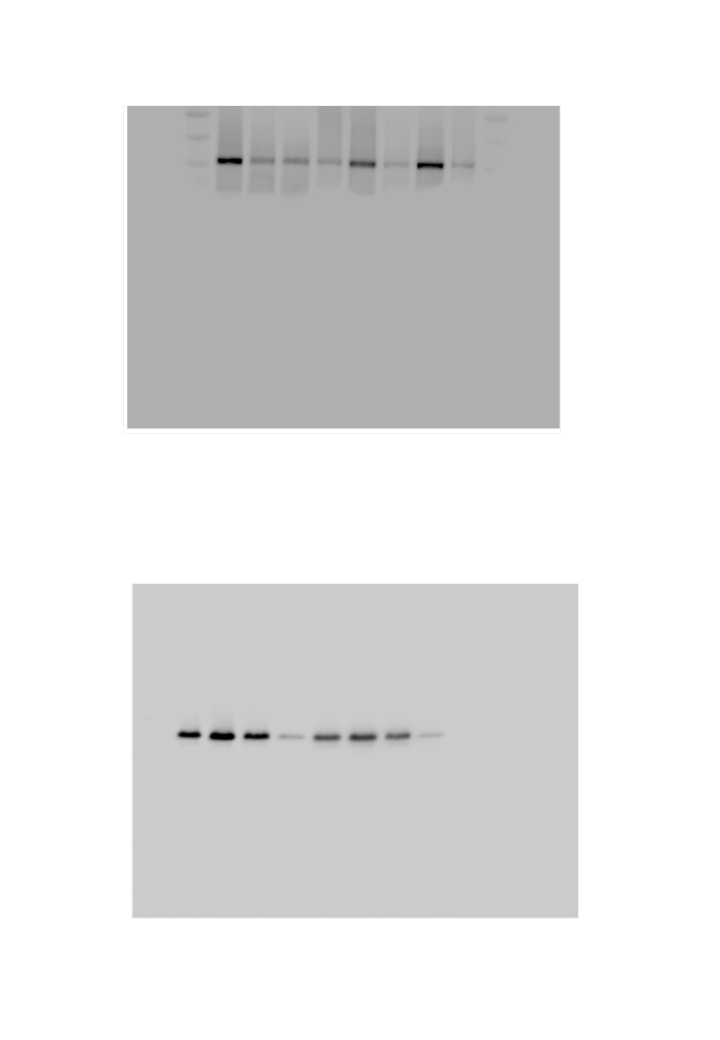

Supplement: Figure 2—source data 4. [file elife-107582-fig2-data4.zip › Figure 2B source data 2/Figure 2B source data HIRA_H3.tiff]

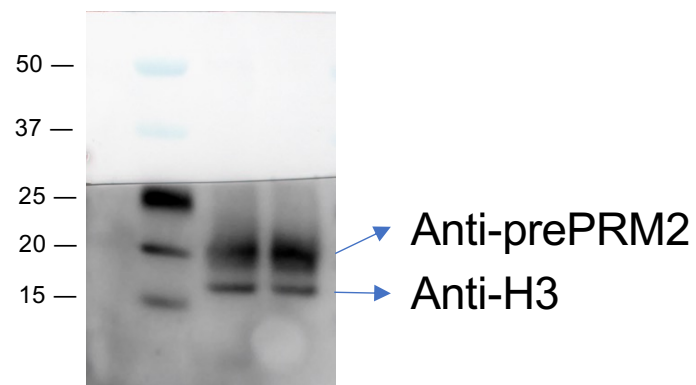

Supplement: Figure 7—figure supplement 1—source data 1. [file elife-107582-fig7-figsupp1-data1.zip › Figure 7-figure supplement 1 source data 1/Figure 7-figure supplement 1 source data1.pdf]

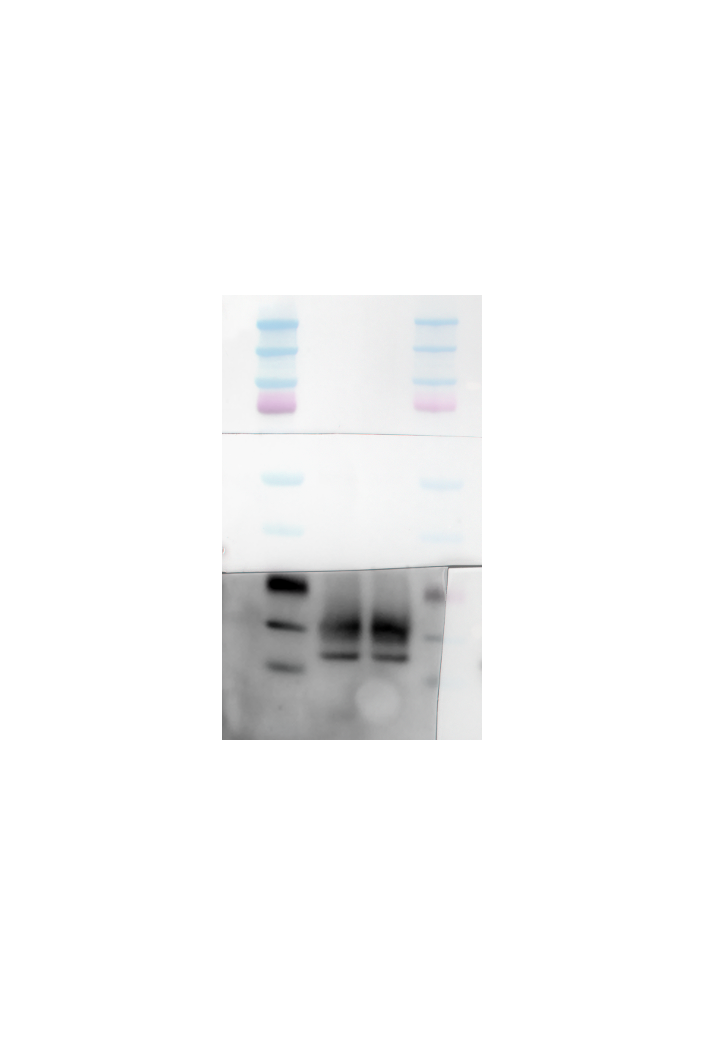

Supplement: Figure 7—figure supplement 1—source data 2. [file elife-107582-fig7-figsupp1-data2.zip › Figure 7-figure supplement 1 source data 2/Figure 7-figure supplement 1 source data pPRM_H3.tiff]
